# P14 Procalcitonin evaluation of antibiotic use in COVID-19 hospitalized patients during the first wave of COVID-19: the PEACH study

**DOI:** 10.1093/jacamr/dlac004.013

**Published:** 2022-02-16

**Authors:** Joanne Euden, Philip Howard, Neil Powell, Martin J. Llewelyn, Tamas Szakmany, Mahableswhar Albur, Stuart E. Bond, Lucy Brookes-Howell, Paul Dark, Thomas P. Hellyer, Susan Hopkins, Iain J. McCullagh, Margaret Ogden, Philip Pallmann, Helena Parsons, David G. Partridge, Dominick E. Shaw, Bethany Shinkins, Stacy Todd, Emma Thomas-Jones, Robert West, Enitan D. Carrol, Jonathan A. T. Sandoe

**Affiliations:** Centre for Trials Research, Cardiff University, Cardiff, UK; University of Leeds, Leeds, UK; Royal Cornwall Hospital Trust, UK; Brighton and Sussex Medical School, University of Sussex, Brighton, UK; The Grange University Hospital, Aneurin Bevan University Health Board, UK; North Bristol NHS Trust, Bristol, UK; Mid Yorkshire Hospitals NHS Trust, UK; Centre for Trials Research, Cardiff University, Cardiff, UK; Manchester NIHR Biomedical Research Centre, University of Manchester, Manchester, UK; Translational and Clinical Research Institute, Newcastle University, Newcastle upon Tyne, UK; Public Health England, UK; The Newcastle upon Tyne hospitals NHS Foundation Trust, Newcastle upon Tyne, UK; Patient and Public Involvement Representative, NIHR, UK; Centre for Trials Research, Cardiff University, Cardiff, UK; Sheffield Teaching Hospitals NHS Foundation Trust, Sheffield, UK; Sheffield Teaching Hospitals NHS Foundation Trust, Sheffield, UK; Division of Respiratory Medicine, University of Nottingham, Nottingham, UK; Leeds Institute of Health Sciences, University of Leeds, Leeds, UK; Liverpool University Hospital NHS Foundation Trust, Liverpool, UK; Centre for Trials Research, Cardiff University, Cardiff, UK; Leeds Institute of Health Sciences, University of Leeds, Leeds, UK; Institute of Infection, Veterinary & Ecological Sciences, University of Liverpool, Liverpool, UK; Leeds Institute of Medical Research, University of Leeds, Leeds, UK

## Abstract

**Background:**

A minority of patients presenting to hospital with COVID-19 have bacterial coinfection. Procalcitonin testing may help identify patients for whom antibiotics should be prescribed or withheld. The PEACH study describes the use of procalcitonin in English and Welsh hospitals during the first wave of the COVID-19 pandemic to help diagnose bacterial infections and guide antibiotic treatment. There is a lack of clear evidence to support its use in lung infections, which means in some hospitals, clinicians have used the procalcitonin test to guide antibiotic decisions in COVID-19, whilst in other hospitals, they have not. Our study is analysing data from hospitals that did and did not use procalcitonin testing during the first wave of the COVID-19 pandemic. It will determine whether and how procalcitonin testing should be used in the NHS in future waves of COVID-19 to protect patients from antibiotic overuse.

**Methods:**

To assess whether the use of PCT testing, to guide antibiotic prescribing, safely reduced antibiotic use among patients who were hospitalized with COVID-19 during the first wave of the pandemic, we are answering this question through three different, and complimentary, work streams (WS), each with discrete work packages (WP): (i) Work Stream 1: utilization of PCT testing to guide antibiotic prescribing during the first wave of COVID-19 pandemic; (ii) Work Stream 2: patient-level impact of PCT testing on antibiotic exposure and clinical outcome (main work stream currently in analysis); and (iii) Work Stream 3: health economics analysis of PCT testing to guide antibiotics in COVID-19.

**Results:**

Our first publication from Work Stream 1 (*Antibiotics* 2021, **10**: 516) used a web-based survey to gather data from antimicrobial leads about the use of procalcitonin testing. Responses were received from 148/151 (98%) eligible hospitals. During the first wave of the COVID-19 pandemic, there was widespread introduction and expansion of PCT use in NHS hospitals. The number of hospitals using PCT in emergency/acute admissions rose from 17 (11%) to 74/146 (50.7%) and use in ICU increased from 70 (47.6%) to 124/147 (84.4%). This increase happened predominantly in March and April 2020, preceding NICE guidance. Approximately half of hospitals used PCT as a single test to guide decisions to discontinue antibiotics and half used repeated measurements. There was marked variation in the thresholds used for empirical antibiotic cessation and guidance about interpretation of values.

**Conclusions:**

Procalcitonin testing has been widely adopted in the NHS during the COVID-19 pandemic in an unevidenced, heterogeneous way and in conflict with relevant NICE guidance. Further research is needed urgently that assesses the impact of this change on antibiotic prescribing and patient safety. Work Stream 2 is ongoing, and results will be published once available.

